# High-frequency conductive hearing loss as a diagnostic test for incomplete ossicular discontinuity in non-cholesteatomatous chronic suppurative otitis media

**DOI:** 10.1371/journal.pone.0189997

**Published:** 2017-12-21

**Authors:** Krishnamurti M. A. Sarmento, André Luiz Lopes Sampaio, Tatiana Guthierre Targino Santos, Carlos Augusto Costa Pires de Oliveira

**Affiliations:** 1 Department of Otolaryngology, Brasilia Military Police Hospital, Brasilia, DF, Brazil; 2 Affiliated Center of the Fisch International Microsurgery Foundation (FIMF), Brasilia, DF, Brazil; 3 Department of Otolaryngology, University of Brasilia, Brasilia, DF, Brazil; University of Palermo, ITALY

## Abstract

Chronic suppurative otitis media, with or without cholesteatoma, may lead to erosion of the ossicles and discontinuity of the ossicular chain. In incomplete ossicular discontinuity (IOD), partial erosion of the ossicles occurs, but some sound transmission is noted throughout the ossicular chain. High-frequency conductive hearing loss (HfCHL) has been considered a hallmark of incomplete ossicular discontinuity. This study aims to evaluate the use of HfCHL as a preoperative predictor of IOD in patients with non-cholesteatomatous chronic suppurative otitis media. The HfCHL test was defined as the preoperative air-bone gap (ABG) at 4 kHz minus the average of the ABG at 0.25 and 0.5 kHz. The test was applied in 328 patients before surgery and compared to intraoperative findings as the gold standard. At surgery, 201 (61.3%) patients had an intact ossicular chain, 44 (13.4%) had a complete ossicular discontinuity, and 83 (25.3%) exhibited an IOD. The best cutoff level was calculated as 10 dB. The HfCHL test to diagnose IOD had a sensitivity of 83% and a specificity of 92% with a post-test probability of 78% and a likelihood ratio of 10.2. We concluded that the HfCHL test is highly effective in predicting IOD in patients with non-cholesteatomatous chronic suppurative otitis media and that it should be used routinely as a screening test prior to surgery.

## Introduction

Chronic suppurative otitis media (CSOM) with or without cholesteatoma may lead to erosion of the ossicles and discontinuity of the ossicular chain [1,2]. This discontinuity may be complete, with no contact between the disconnected ends, or incomplete, with sound and movements partially transmitted. Preoperative findings may suggest either a complete or an incomplete ossicular discontinuity, but the status of the ossicular chain can only be truly identified intraoperatively [3].

**Fig 1:**
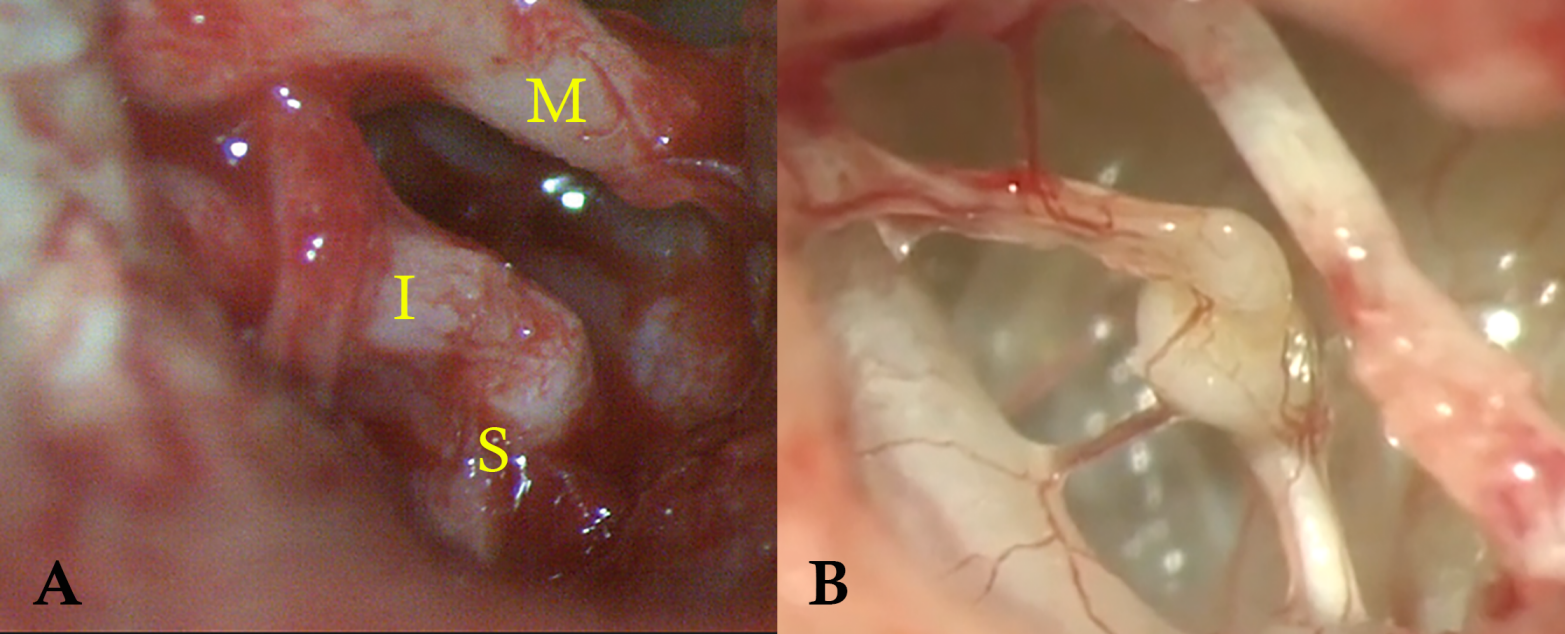
Incomplete ossicular discontinuity. A: IOD type 1. There is erosion of the long process of the incus but still bony contact between incus and stapes. (I) incus, (M) malleus and (S) stapes. B: IOD Type 2. Incus and stapes are connected mainly by soft tissue.

Incomplete ossicular discontinuity (IOD) can be further classified into type 1, when there is still bone-to-bone interaction, and type 2, when the eroded ossicles are connected mainly by soft tissue (Fig 1). Our previously published data indicates that IOD type 1 may not need intervention whereas type 2 IOD will result in a less than desirable hearing result if not treated with ossicular reconstruction [4].

Complete ossicular discontinuity typically results in an audiometric pattern of a large, flat conductive hearing loss. In contrast, high-frequency conductive hearing loss (HfCHL) has been described as a hallmark of incomplete ossicular discontinuity [5,6]. This is because the ossicular defects found in IOD are believed to transmit low-frequency sounds more effectively than high-frequency sounds, resulting in a larger air-bone gap (ABG) in high frequencies and a rather unusual pattern of a down slope audiogram with preserved bone conduction.

In patients with IOD and an intact tympanic membrane (after trauma, for example, or after a tympanoplasty in which the ossicular chain defects failed to be identified or corrected), the triad of HfCHL, fluctuating hearing and short-term improvement in hearing with the Valsalva maneuver is commonly present [5]. Patients with suppurative chronic otitis media and complete ossicular discontinuity exhibit more conductive hearing loss compared with patients with an intact ossicular chain, regardless of the size of the perforation. Nevertheless, no identifiable studies have investigated the presence of HfCHL in patients with chronic otitis media prior to surgery. If HfCHL could help to identify patients with IOD before surgery, it would serve as a very useful diagnostic tool.

This study aims to evaluate HfCHL as a diagnostic test for the presence of incomplete ossicular discontinuity in patients with non-cholesteatomatous CSOM.

## Patients and methods

We conducted a prospective study over an 8-year period at a tertiary reference hospital. The Bonsucesso Federal Hospital medical ethical committee approved this study and all patients were treated according to the protocol. Patients gave their written informed consent to participate in the study.

All patients with chronic suppurative otitis media who submitted to a tympanoplasty were consecutively included until the minimum required sample size was reached. The ossicular chain was inspected and its configuration registered in all cases. Patients with previous otologic surgery on the same ear were excluded. Since the relevant findings for this study were the preoperative audiogram and the intraoperative ossicular chain configuration, there was no need to exclude patients based on their postoperative outcomes.

We calculated the minimum sample size for validating this new diagnostic test as 310 subjects for an anticipated test sensitivity of at least 80% with a precision of 0.03 and a 95% confidence level based on previously published methodology [7,8].

Patients were subject to a hearing test no longer than 3 months prior to surgery. We defined the HfCHL test in a similar manner as described by Sim et al. [9]: the ABG was set at 4 kHz minus the mean ABG at 0.25 and 0.5 kHz.

HfCHLTest=ABG4kHz–[(ABG0.25kHz+ABG0.5kHz)/2]

The test results were compared with the gold standard of intraoperative findings. Based on surgical findings, patients were classified as having an intact ossicular chain, complete ossicular discontinuity or incomplete ossicular discontinuity (further subdivided into type 1 and 2 IOD). As the purpose of this study was to evaluate the diagnostic value of the HfCHL test in identifying patients with IOD, two scenarios were considered. In the first, the target group for the test (disease group) included all patients with IOD. In the second scenario, only patients with type 2 IOD (soft tissue band bridging ossicles) were included in the disease group. Every calculation described below was repeated for both scenarios.

Data entry was performed using SAS 6.11 software (SAS Institute, Inc., Cary, North Carolina).

Association of test results with IOD was analyzed using univariate logistic regression analysis.

Pre-test probability was calculated according to the prevalence of the “disease” in each scenario. Sensitivity, specificity, post-test probability and likelihood ratios were calculated for each possible cutoff value of the test, ranging from -5 to +47.75 dB at 0.25-dB increments. The plot of sensitivity versus 1-specificity was generated, and the receiver operating characteristic (ROC) curve was drawn. The best cutoff value was derived from the ROC curve analysis and the area under the curve (AUC). The statistical significance of differences in areas under the ROC curve (AUC) between scenarios was determined using a nonparametric method [10]. The statistical significance of differences in sensitivity and specificity levels was investigated using McNemar's test for paired proportions. The results are also presented using Bayes’ nomogram to facilitate interpretation.

## Results

A total of 328 patients with CSOM without cholesteatoma were tested for HfCHL preoperatively and had their intraoperative findings recorded (gold standard). Age varied from 18 to 62 years with a mean age of 35.7 (±12.4 SD). There was a homogeneous distribution between genders.

Surgery revealed that 201 (61.3%) patients had an intact ossicular chain, 44 (13.4%) had complete ossicular discontinuity and 83 (25.3%) were exhibited incomplete ossicular discontinuity, which was further divided in 48 type 1 IOD (14,6%) and 35 type 2 IOD (10.7%) cases.

**Fig 2:**
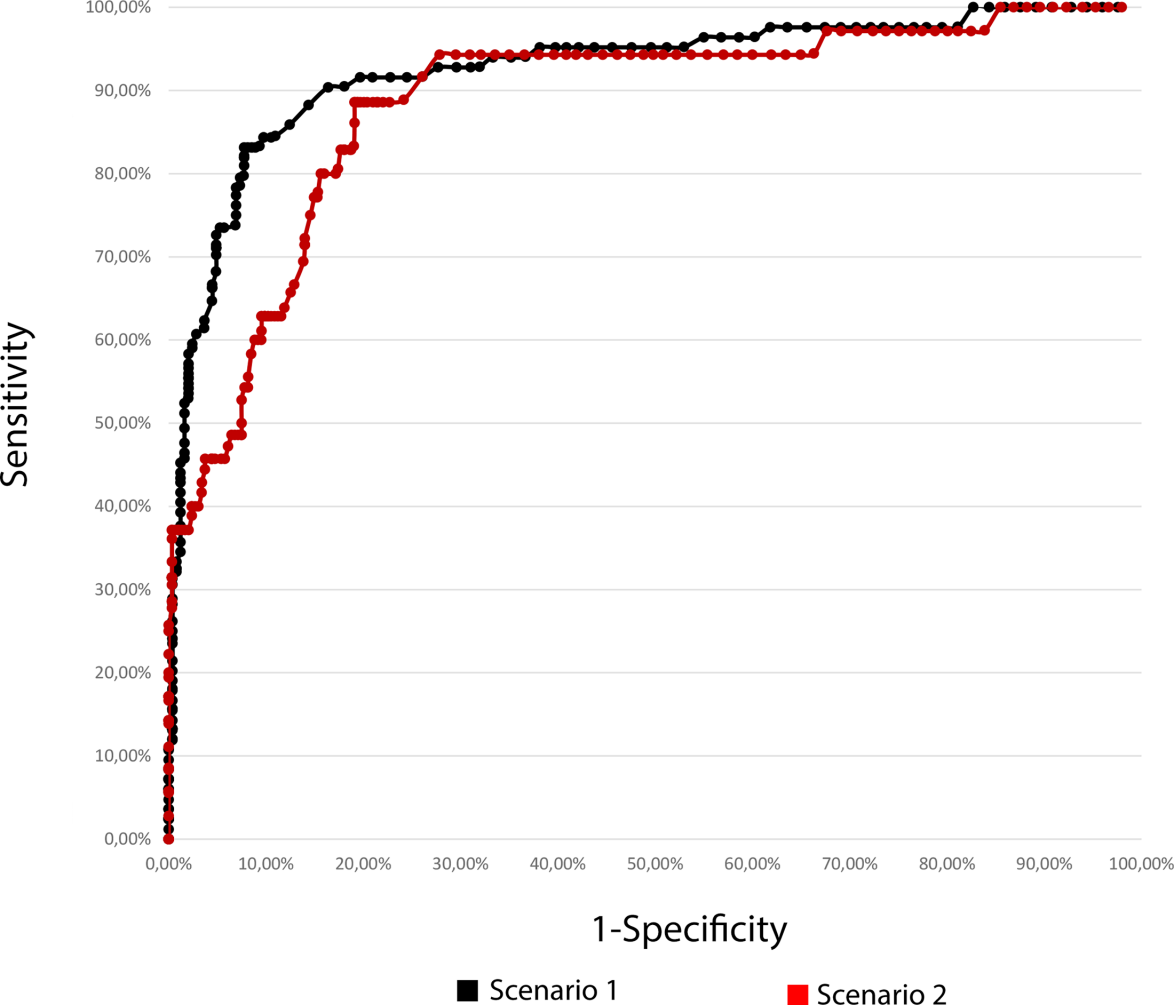
ROC curves for scenarios 1 and 2. Receiver operating characteristics curves (n = 328) for the HfCHL test. Scenario 1 (in black): Target group for the test (disease group) included all patients with incomplete ossicular discontinuity. Scenario 2 (in red): Target group for the test (disease group) exclusively included patients with type 2 IOD.

Sensitivity and specificity were calculated for each possible test result cutoff point in each scenario (as explained in Methods). Fig 2 presents the ROC curves obtained.

In both scenarios, the best cutoff point for the HfCHL test was 10 dB. Table 1 presents the main properties of the test in each scenario.

**Table 1:** HfCHL test properties.

	Scenario 1 (Disease group = All IOD)	Scenario 2 (Disease group = Type 2 IOD)
Cutoff value for HfCHL test	10 dB	10 dB
Sensitivity	83%	89%
Specificity	92%	80%
Pre-test probability	25%	11%
Post-test probability	78%	35%
Likelihood ratio	10.2	4.5
AUC (SE)	0.901 (0.019)	0.865 (0.026)

IOD: Incomplete Ossicular Discontinuity; HfCHL: High-frequency Conductive Hearing Loss; AUC: Area Under the receiver operating characteristics (ROC) Curve; SE: Standard Error.

**Fig 3:**
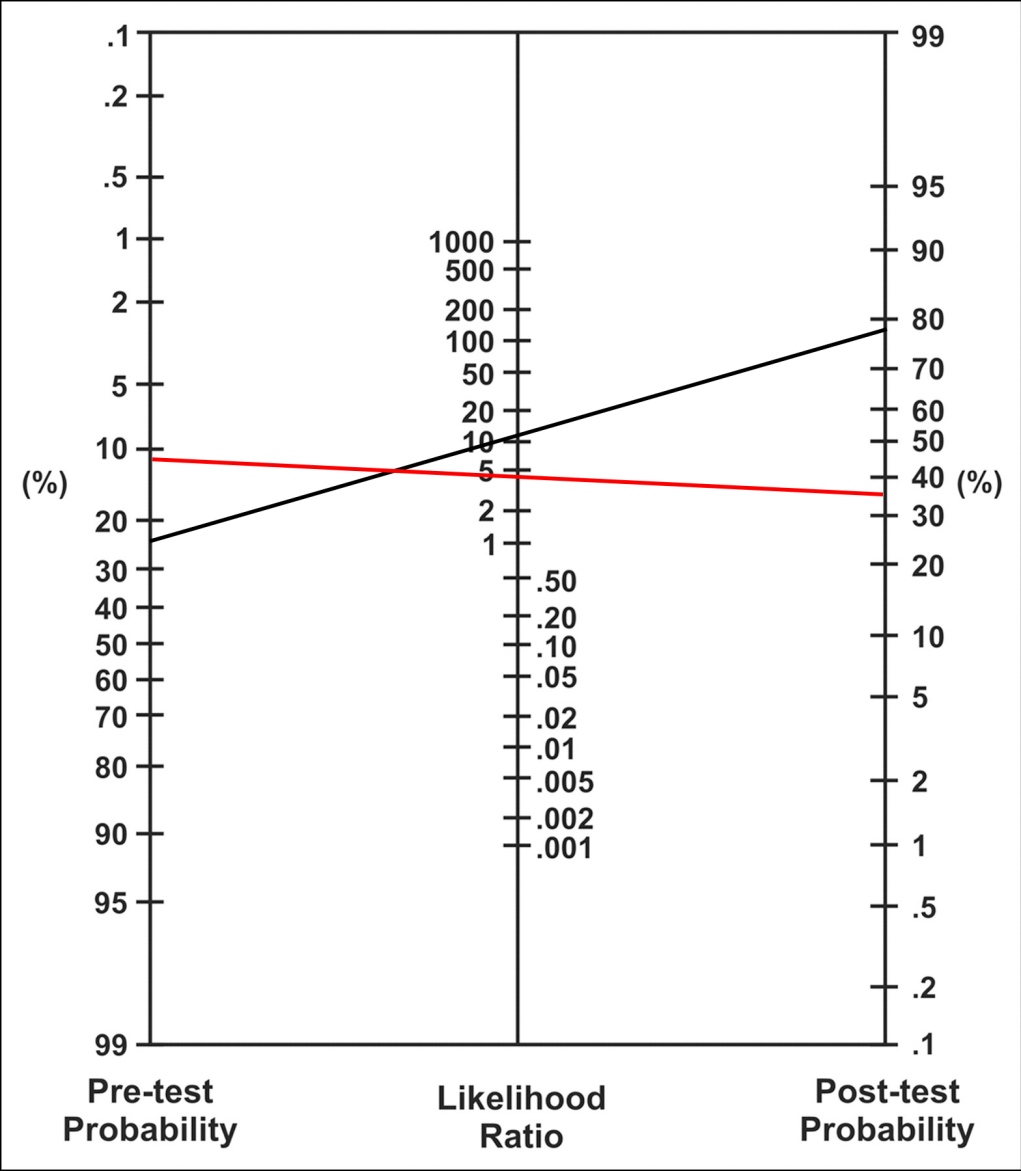
Bayes’ nomogram for scenarios 1 and 2. The test performance for scenario 1 is indicated in black, and that for scenario 2 is indicated in red.

Bayes’ nomogram is presented in Fig 3 to facilitate interpretation of the HfCHL test as a diagnostic tool. Our results indicate a better performance of the test in scenario 1.

## Discussion

Although the existence of partial ossicular erosion causing incomplete ossicular discontinuity in chronic suppurative otitis media has been acknowledged for over 120 years [11], the subject has been greatly neglected in the literature. To a large extent, this lack of attention is attributed to the fact that current and past classifications of ossicular defects in CSOM do not take into account partial erosions, classifying the ossicles as either present or absent [12,13]. As a consequence, authors tend to record intraoperative findings and report their results in the same fashion, overlooking partial erosions and IOD.

Beickert was the first to describe the pattern of high-frequency conductive hearing loss and its relation to an IOD [5]. He presented the condition as part of a triad that also included fluctuating hearing loss and short-term improvement in hearing with the Valsalva maneuver. According to Beickert, the bulging of the ear drum, which is caused by the increased air pressure in the middle ear with the Valsalva maneuver, would strain the soft tissues embedded within the partially eroded ossicular chain and briefly improve sound conduction. However, in the resting position, the eroded areas, which are replaced by scar and granulation tissues, are more elastic than solid bone and tend to dissipate high-frequency sound waves instead of transmitting them, resulting in HfCHL.

Anderson and Barr revisited the subject in 1971, describing HfCHL in 22 patients and confirming some type of IOD at surgery in 14 of the patients [6]. Another few case reports of temporal bone traumas and a resulting HfCHL were also published in the following decades [14,15].

More recently, Sim et al. studied 14 patients who were submitted to tympanoplasty with successful closure of the tympanic perforation but had to undergo another operation due to bad functional results. All of these patients presented with IOD at the second surgery. Thirteen of these patients had HfCHL, and 10 presented with the complete Beickert’s triad before the second surgery. The authors complemented their study with a mathematical model that could explain the high-frequency hearing loss caused by IOD.

Finally, Farahmand et al. reviewed the surgical notes of 66 patients submitted to exploratory surgery with either complete (26 cases) or incomplete ossicular discontinuity (40 cases) and an intact tympanic membrane [16]. The intraoperative findings were compared to the preoperative audiometry, and only 8 of the 66 patients had HfCHL, 7 of whom had IOD. Nevertheless, the authors used a different formula to calculate HfCHL: the mean ABG of 2 and 4 kHz minus the mean ABG of 0.25 and 0.5 kHz. In our opinion, the use of the 2 kHz frequency (not previously described) might have negatively influenced their results, since it substantially reduced the resulting difference calculated by the test. Furthermore, the authors classified cases as complete or incomplete ossicular discontinuity retrospectively based on surgical notes that they admitted were not always clear regarding the ossicular chain configuration.

All of the aforementioned authors have studied HfCHL in patients with an intact tympanic membrane. The patients represent cases of traumatic lesions to the ossicles or were patients previously submitted to a tympanoplasty in which the ossicular chain defects persisted. Although identifying IOD in these cases could be useful, the study is limited to a rather small number of cases who need a second intervention. The clinical applicability would be considerably increased if the test could predict ossicular discontinuity before the primary surgery, when the patient still has a tympanic perforation.

Carrillo et al. published a study aiming to identify preoperative audiometric patterns in patients with chronic otitis media that could predict the presence of complete ossicular discontinuity before primary surgery [17]. The authors noted a strong correlation between ABG greater than 40 dB at 4 kHz and complete ossicular discontinuity. However, the authors did not mention the IOD situation nor did they study HfCHL.

Multi-frequency tympanometry has also been used to predict the diagnosis of various middle ear pathologies preoperatively, including ossicular discontinuity, as changes in mass and/or stiffness of the mechano-acoustic system of the middle ear are found to affect the resonant frequency. Wada et al. reported that a discontinuity in the ossicular chain (surgically confirmed in 84% of their cases) results in lower resonant frequency values, by decreasing the stiffness in the middle ear [18].

To the best of our knowledge, our study was the first to prospectively analyze HfCHL as a diagnostic tool for IOD in patients with CSOM before primary surgery and compare this procedure with the best possible gold standard, namely, intraoperative findings. Although surgical findings might not be conclusive on the presence of IOD as differentiating partial versus complete discontinuity can occasionally be difficult based on its location and tissue appearance, surgery remains the best possible method of verifying the condition. In addition, as this was a prospective study, the surgeon was actively assessing the ossicular chain status and describing it accurately and in detail in the study protocol in contrast to using general surgical notes.

We were also the first study with a sufficiently large series to provide proper statistical power to our analysis in determining the test properties. Finally, this was the first study on the subject to test patients with CSOM consecutively regardless of their ossicular status. Using this study design, we could establish the prevalence of each ossicular situation (intact, partially eroded or discontinued) in the tested population, which is essential to calculate pre- and post-test probabilities.

Whenever a diagnostic test is evaluated, it is paramount to define the “sick” and the “healthy” subjects, so the test results can be compared with the “truth” (or what the gold standard states as true). In many cases, even when a trustworthy gold standard is available, defining sick and healthy can be tricky because borderline cases are always present. In our study, we utilized two different scenarios. In the first scenario, all cases of incomplete ossicular discontinuity were deemed “sick”. In the second scenario, only cases of type 2 IOD were included in the “sick” group, and all type 1 IOD cases were included in the healthy group.

Type 2 IOD occurs when ossicular erosion is so extensive that bone-to-bone contact no longer exists and ossicles are connected only (or mainly) by soft tissue. This scenario represents a more advanced stage of erosion compared with type 1 IOD, which could be subsequently considered “closer to normal” or “borderline”. Furthermore, one of our previous publications showed that, in type 2 IOD, ossicular reconstruction has better hearing results than no reconstruction at all; whereas in type 1 IOD, the functional results between incus interposition and no reconstruction were similar [4].

Thus, in the first scenario, borderline cases were included in the sick group, whereas these cases were excluded from the sick group and consequently considered healthy in the second scenario.

Sensitivity measures the proportion of positives that are correctly identified as such (i.e., the percentage of sick people who are correctly identified by the test as having the condition). Specificity measures the proportion of negatives that are correctly identified as such (i.e., the percentage of healthy people who are correctly identified by the test as not having the condition). If the borderline cases are considered as sick (scenario 1), there is a greater chance that the test will fail to identify sick people as sick (worse sensitivity) and a better chance of the test identifying healthy people as healthy (better specificity). The opposite occurs when the borderline cases are shifted to the healthy group, as in scenario 2 (better sensitivity and worse specificity). In this study, the sensitivity and specificity were 83% and 92%, respectively, for the first scenario and 89% and 80%, respectively, for second scenario. Thus, the differences between scenarios in these two parameters were as expected.

The HfCHL test could be considered a screening test given that its purpose is to alert the surgeon to a possible need for ossicular reconstruction. Although we strongly feel that the ossicular chain should be inspected in all tympanoplasties, many surgeons will not do it when operating on a non-cholesteatomatous case, especially with a smaller perforation and if the mucosa of the mesotympanum appears normal. If a test could predict an increased chance of an ossicular defect, inspection of the ossicular chain would be mandatory in these cases, thus improving the hearing outcome among patients overall. Furthermore, if the need for an ossicular reconstruction is anticipated, the surgeon can plan in advance and make necessary arrangements, such as schedule a longer time at the operation theater, have the prosthesis and other ossicular reconstruction materials available, and set up the drilling system.

Considering IOD cases are borderline by definition, there is also the possibility of the surgeon remaining in doubt even after inspecting the ossicular chain. In patients with larger tympanic perforations, it is not always clear whether the preoperative air-bone gap is entirely due to the perforation or worsen by an associated ossicular defect. If one inspects the ossicular chain and finds some degree of erosion but an apparently functional ossicular chain, the HfCHL test can be another factor to be weighted in the decision-making process of reconstructing it or not.

As a screening test, it is more logical to include borderline cases in the disease group than in the healthy group given that the idea is not to miss any cases in which the ossicular chain should be thoroughly inspected. Thus, the results of the first scenario are more important regarding the clinical applicability of the test.

Sensitivity and specificity were calculated for each possible cutoff level, and the best cutoff was chosen based on the receiver operating characteristic (ROC) curve. The best cutoff was 10 dB for both scenarios. Interestingly, this was the same cutoff level used in previous studies, where it was defined intuitively [9,16].

When the ROC curve is used, the area under the curve (AUC) is another parameter that can be derived. The area under a ROC curve measures discrimination, which is the ability of the test to correctly classify those with and without the disease. Simply put, the AUC combines sensitivity and specificity into a single parameter. An AUC value between 0.7 and 0.8 is considered fair, between 0.8 and 0.9 is good, and greater than 0.9 is excellent. The HfCHL test AUC values for scenarios 1 and 2 were 0.901 and 0.865, respectively.

Of note, patients who exhibit complete ossicular discontinuity at surgery were grouped with patients with an intact ossicular chain in the “healthy” group. This classification is because the HfCHL test aims to differentiate incomplete ossicular discontinuity from the other cases regardless of whether both normal and complete ossicular discontinuity are included in the “rest”. Patients with a complete ossicular discontinuity generally have a larger air-bone gap compared with patients with a normal ossicular chain [17]. However, given that the HfCHL test calculates the differential between the ABG of higher and lower frequencies, the absolute values of the ABG are irrelevant.

Although clinicians are accustomed to the terms “sensitivity” and “specificity” as measures of a diagnostic test’s reliability, these test characteristics exhibit certain disadvantages. These terms pertain to the likelihood of a particular test result in patients independently known to have or not to have the disease in question. These terms do not inform the clinician how likely the individual patient is to have the condition as a result of that test result. The magnitude of change from a clinician’s initial (pretest) assessment of the probability of disease to the likelihood of disease after knowing the result of a diagnostic test (posttest probability) is represented by the likelihood ratio (LR) [19].

The LR of the HfCHL test in the first scenario is 10.2, indicating that the HfCHL test is ten-fold more likely to be positive in patients with incomplete ossicular discontinuity compared with patients without IOD. This information is more clinically applicable compared with the isolated sensitivity and specificity parameters. A diagnostic test is generally considered as high impact if the LR value is greater than 10 [20].

Another method to assess at a test’s efficacy involves pre- and post-test probabilities. The Bayes’ nomogram presented in Fig 2 is a visual tool that demonstrates the clinical impact of the test. In scenario 1, the pre-test probability (the chance of a patient having IOD) is equal to the condition’s prevalence in the population, which was 25%. The post-test probability is 78%. Thus, if the HfCHL test result is greater than 10 dB (positive), there is a 78% chance that the patient will have an IOD. A post-test probability close to 80% is considered high [20].

## Conclusions

High-frequency conductive hearing loss (defined as the preoperative ABG at 4 kHz minus the average of the ABG at 0.25 and 0.5 kHz) can be used as a diagnostic test for incomplete ossicular discontinuation in patients with non-cholesteatomatous chronic suppurative otitis media before primary surgery.

Patients with a test result greater than 10 dB are 10-fold more likely to have IOD compared with patients with a test result less than 10 dB. The sensitivity and specificity of the test were 83% and 92%, respectively, when all patients with IOD were considered and 89% and 80%, respectively, when only type 2 IOD cases were considered.

## Supporting information

S1 Spreadsheet Raw data of the audiograms. (XLSX)
